# Diagnostic and Prognostic Values of BMPER in Patients with Urosepsis following Ureteroscopic Lithotripsy

**DOI:** 10.1155/2019/8078139

**Published:** 2019-01-20

**Authors:** Chang Geng Xu, Yong Lian Guo

**Affiliations:** Department of Urology, The Central Hospital of Wuhan, Tongji Medical College, Huazhong University of Science and Technology, Wuhan, 430014, China

## Abstract

The present study aims to investigate the risk factors for urosepsis and the diagnostic and prognostic values of the bone morphogenetic protein endothelial cell precursor-derived regulator (BMPER) in patients with urosepsis following ureteroscopic lithotripsy. A total of 305 patients with unilateral ureteral obstruction caused by calculi were included in the study. Patients were divided into three groups, namely, high, medium, and low perfusion pressure groups. The serum C-reactive protein, procalcitonin, lactate (LAC), and BMPER were measured after operation. A logistic regression model was used to assess the risk factors for postoperative urosepsis. The relationships of BMPER with laboratory parameters were explored with a multiple linear regression model. Receiver operating characteristic (ROC) curves were used to diagnosis urosepsis. The cumulative incidence of the adverse events after operation was calculated and compared by log-rank test. Forty-five patients (14.8%) had an episode of urosepsis after operation. Irrigation pressure was an independent risk factor for urosepsis. LAC and sequential organ failure assessment (SOFA) were associated with BMPER after operation. The area under curve value of BMPER for urosepsis was 0.829 (95% confidence interval [CI], 0.773 to 0.884). Uroseptic patients with higher BMPER concentration exhibited more adverse outcome. BMPER possesses valuable discriminative capacity for urosepsis and is a strong predictor of adverse outcome in patients with urosepsis.

## 1. Introduction

Urosepsis accounts for approximately 25% of all sepsis cases and always develops from complicated urinary tract infection, which is often secondary to urinary tract obstruction, such as ureteral calculi and stenosis. Ureteroscopic lithotripsy (URSL) is the most frequently used technique for ureteral stone treatment [1]. Postoperative urinary infection is one of the most common complications of the procedure. Urosepsis is the most serious infection type that can result in shock and sepsis-related death, thereby indicating the importance of rapid and accurate diagnosis and proper treatment [2]. A wide range of biomarkers were evaluated previously, but only few showed sufficient sensitivity or specificity to reliably diagnose and predict the future course of patients with urosepsis [3]. Therefore, the evaluation of new diagnostic biomarkers discriminating patients with sepsis or not in an early stage is essential.

Bone morphogenetic protein endothelial cell precursor-derived regulator (BMPER) was originally identified in a screen for differentially expressed proteins in embryonic endothelial precursor cells [4]. Several studies have shown that BMPER plays an important role in vascular endothelial inflammation [5–9]. Under chronic injury condition, statins can upgrade the BMPER level in endothelial cells, and BMPER plays an anti-inflammatory role by downregulating intercellular adhesion molecule-1 [5]. BMPER also inhibits the endothelial expression of inflammatory adhesion molecules and protects against atherosclerosis [7]. BMPER knockdown potentiates TNF*α*-induced endothelial inflammatory responses [6]. This anti-inflammatory phenotype of BMPER is mediated by blocking the BMP activity. By contrast, in acute inflammatory response, BMPER exerts a proinflammatory feature via nuclear factor of activated T cells-1 activation, which is initiated by BMPER/low-density lipoprotein receptor-related protein 1 interaction [9]. In terms of the important role of endothelial injury and inflammation in sepsis development, BMPER has considerable potential as a novel early diagnostic and prognostic biomarker in infectious diseases [10]. Accordingly, we hypothesized that the serum BMPER levels were different between patients with urosepsis and those without, and its changes can predict the course of patients.

Sepsis definitions were recently updated and published in the Third International Consensus Definitions for Sepsis and Septic Shock (Sepsis3) [11]. Studies on the biomarkers in evaluating the value of BMPER according to the latest Sepsis-3 definitions in urologic setting are unavailable [12]. Therefore, the aim of this study was to evaluate the diagnostic and prognostic values of BMPER in patients with urosepsis following URSL.

## 2. Materials and Methods

This retrospective, case-control study was conducted in the Wuhan Central Hospital of Huazhong University of Science and Technology Tongji Medical College from July 2013 to February 2018. The study was approved by the local ethics committees, and all included patients provided written informed consent.

A total of 85 healthy control subjects and 305 patients with unilateral ureteral obstruction (UUO) caused by calculi were included in the study. Individuals without any known illness presenting to the outpatient department for routine check-up were selected as the control group. The inclusion criteria for patients with UUO are as follows: patients with unilateral ureteral stone without urinary tract infection by urine analysis and culture (absence of pyuria and bacteriuria). The exclusion criteria for subjects with UUO are as follows: patients with active infection, patients requiring second operation due to large residual stone, immunocompromised, patients with renal insufficiency, solitary kidney, patients with congenital urinary tract anomalies, and patients with other diseases (e.g., pulmonary disease, liver disease, essential hypertension, and cardiovascular disease). Routine laboratory studies and image examinations were conducted before URSL. The diagnosis of UUO was based on clinical manifestations, urinary sediment, plain X-ray findings, intravenous pyelonephrography, ultrasound, or computed tomography.

The collected variables are as follows: age, gender, body mass index (BMI), medical history, stone laterality, stone site, stone size, hydronephrosis, operation time, white blood cell (WBC), body temperature (T), heart rate (HR), mean arterial pressure (MAP), platelet (PLT), C-reactive protein (CRP), procalcitonin (PCT), BMPER, and creatinine (Cr).

The operation was performed in the lithotomy position under general anesthesia by an experienced surgeon. Ureteroscopy was performed using a Storz rigid ureteroscope. To investigate whether urosepsis is related to the irrigation pressure of URSL, we subjected all patients with UUO to URSL with the irrigation pressure of 60 (low irrigation pressure group), 80 (medium irrigation pressure group), and 100 mmHg (high irrigation pressure group). The irrigation pressure was selected based on the interoperative situation, as follows: low perfusion pressure was selected first. If ureter stenosis was encountered or the vision was unclear due to lithotripsy during operation, then medium or high perfusion pressure was selected. All patients undergoing Holmium: YAG laser lithotripsy possessed a 4.7 or 6 Fr double-J stent and a Foley catheter placed at the end of the procedure. The indwelling Foley catheter was drawn within 48 h. Double-J stent was drawn within 1 month. All patients that are symptomatic or signs of potential sepsis were present within 24 h after URSL. These patients fulfilled the criteria of Sepsis-3 [13]. Patients who developed sepsis were treated with antimicrobial agents. Intensive medical treatment should be instigated if needed.

Blood samples for biomarker measurements were taken at admission, within 24 h after URSL for nonseptic subjects or septic patients, 5 days after URSL for septic patients only. Blood samples were collected from an indwelling arterial or venous catheter, with anticoagulant (ethylenediamine tetra acetic acid) or without anticoagulant. Then, these samples were centrifuged at 3,000 rpm for 10 min at 4°C and the serum/plasma was immediately frozen and stored at −80°C until the final analysis.

WBC and PLT counts were measured using a hematology analyzer (Xuzhou Forward Medical Instrument, Jiangsu, China). The Cr, serum albumin, and bilirubin levels were measured using a biochemistry analyzer (Mindray, Shenzhen, China). The biochemical parameters were measured by routine methods with commercial kits. The CRP level was measured using an immunoturbidimetric assay (Modular Analytics P, Roche Diagnostics, Mannheim, Germany). The LAC level was determined using enzymatic method (Techno Medica, Yokohama, Japan), and the PCT level was evaluated using an immunoluminometric assay (Brahms Diagnostica, Berlin, Germany).

The serum BMPER levels were measured with ELISA kits (CUSABIO, Houston, USA) in accordance with the manufacturer's instructions. The intra-assay coefficients of variation were < 8% and the inter-assay coefficients of variation were < 10%. All assays were measured in duplicate aliquots, and BMPER concentrations were expressed as ng/mL.

All continuous data were expressed as mean ± SD when the data were normally distributed or median (interquartile range) when the data showed skewed distribution. Deviations from a Gaussian distribution were tested by the Kolmogorov-Smirnov test. Continuous variable comparisons were performed with ANOVA or Kruskal-Wallis test. For repeated measured data, Friedman test was applied. Multiple comparisons were evaluated with ANOVA followed by Student-Newman-Keuls test. Categorical variables were assessed by Chi-squared or Fisher's exact test. Spearman rank correlation for nonparametric data was used to test the association of BMPER levels with medical parameters. Multiple logistic regression analysis was used to evaluate the risk factors for urosepsis. In logistic regression, groups were set as dummy variables (low pressure irrigation as reference), and variables in the model were age, gender, diabetes mellitus (DM), BMI, stone laterality, stone site, stone size, degree of hydronephrosis, irrigation pression, and operation time. The variables were selected with the forward-likelihood ratio. Multiple liner regression analysis was used to analyze significant factors for BMPER. The four variables in the model were CRP, PCT, LAC, and sequential organ failure assessment (SOFA). Receiver operating characteristic (ROC) curves were used to diagnose urosepsis and determine the cut-off values. Uroseptic patients were divided into two groups on the basis of the median BMPER level. The cumulative incidence of the adverse events was defined as death or readmission due to febrile urinary tract infection during the 90 day follow-up. The cumulative incidence of the adverse events was compared by log-rank test. The data was analyzed with SPSS, version 19.0 (SPSS Inc., Chicago, IL, USA). A two-sided* P* < 0.05 was considered statistically significant.

## 3. Results

The baseline characteristics of the study groups are shown in Table 1. There were no significant differences in age, sex distribution, diabetes ratio, BMI, and serum concentrations of CRP, PCT, BMPER, Cr, serum albumin, and bilirubin. There were also no significant differences in T, HR, MAP, WBC, and PLT counts among the four groups. No significant differences were observed in stone side distribution, stone site, stone size, degree of hydronephrosis, and operation time among the three UUO subgroups.

**Figure 1:**
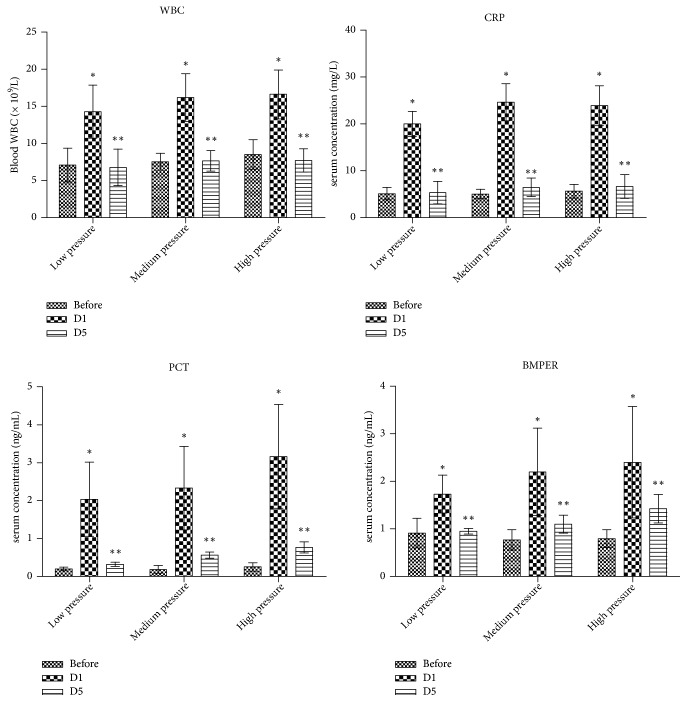
Parameters of patients with urosepsis before treatment and 1 and 5 days after URSL. WBC: white blood cell; CRP: C-reactive protein; PCT: procalcitonin; BMPER: BMP-binding endothelial cell precursor-derived regulator. Before: before operation; D1: 1 day after operation; D5: 5 days after operation; URSL: ureteroscopic lithotripsy. *∗*The parameters were compared between 1 day after operation and before operation; *∗∗* the parameters were compared between 1 and 5 days after operation (*P* < 0.05)

After URSL, the uroseptic rates of the three UUO subgroups were 7.1% (8/112), 14.6% (15/103), and 24.4% (22/90), thereby indicating that the increased incidence of urosepsis was accompanied by the increased irrigation pressure. As shown in Figure 1, WBC counts and the serum CRP, PCT, and BMPER concentration all increased 1 day after ureteroscopy (*P* < 0.05). These parameters all decreased 5 days after ureteroscopy followed by antibiotics treatment (*P* < 0.05).

At 1 day after URSL, there were 31 urosepsis patients and 14 uroseptic shock patients (Table 2). The risk factors for urosepsis were analyzed. Univariate analysis showed that DM, operation time, and irrigation pressure were associated with urosepsis. Multiple logistic regression analysis confirmed the results and revealed that operation time [odds ratio (OR) =1.129, 95% confidence interval (CI), 1.042 to 1.223, and* P* = 0.003], DM (OR = 8.487, 95% CI, 3.549 to 20.294, and* P* < 0.001), and high irrigation pressure (OR = 3.410, 95% CI, 1.262 to 9.212, and* P* = 0.016) were the independent risk factors for urosepsis, when age, sex, BMI, stone side, stone site, stone size, and hydronephrosis were considered (Table 3). The concordance-index was 0.788 (95% CI, 0.727 to 0.849), which indicated good discrimination of the model. The *P* value of calibration is 0.777 by the Hosmer-Lemeshow good of fit test.

We explored the relationship of BMPER with laboratory parameters in patients with urosepsis at 1 day after URSL. As shown in Table 4, the serum BMPER concentration was positively correlated with the serum CRP, PCT, and LAC concentrations and SOFA, while the other laboratory parameters were unrelated to BMPER. Stepwise multiple liner regression analysis showed that the significant factors for BMPER were LAC and SOFA (Table 5).

**Figure 2:**
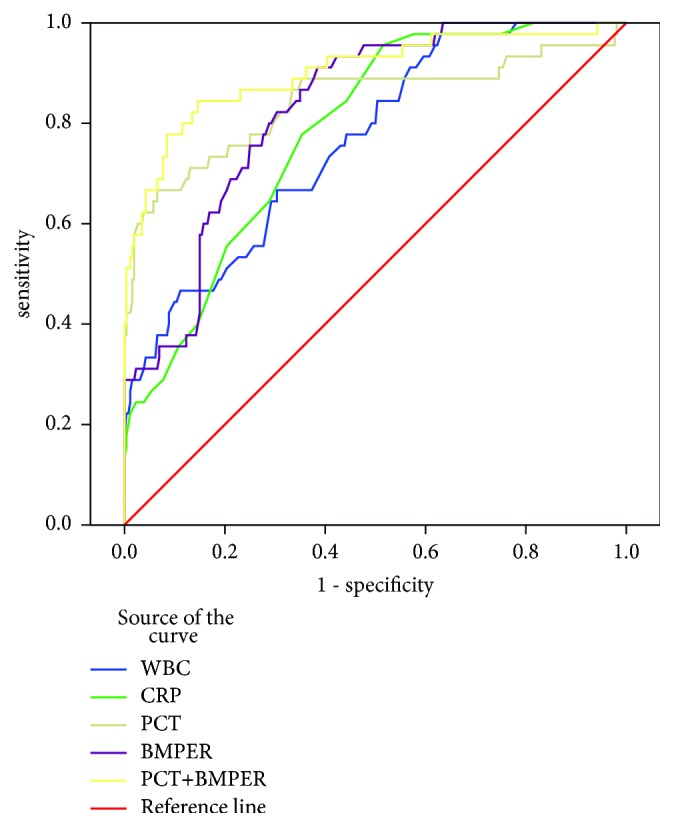
ROC curves of WBC, CRP, LAC, PCT, BMPER, and PCT combined with BMPER for URSL-induced urosepsis diagnosis in patients with UUO. WBC: white blood cell; CRP: C-reactive protein; PCT: procalcitonin; BMPER: BMP-binding endothelial cell precursor-derived regulator; URSL: ureteroscopic lithotripsy; UUO: unilateral ureteral obstruction.

The ROC curves of WBC, CRP, LAC, PCT, BMPER, and PCT combined with BMPER for URSL-induced urosepsis in patients with UUO are shown in Figure 2. The area under curves (AUCs) is listed in Table 6. The AUC value of BMPER was 0.829, which was higher than those of WBC (0.762) and CRP (0.784) but lower than that of PCT (0.843). The AUC value was 0.901 when BMPER was combined with PCT.

**Figure 3:**
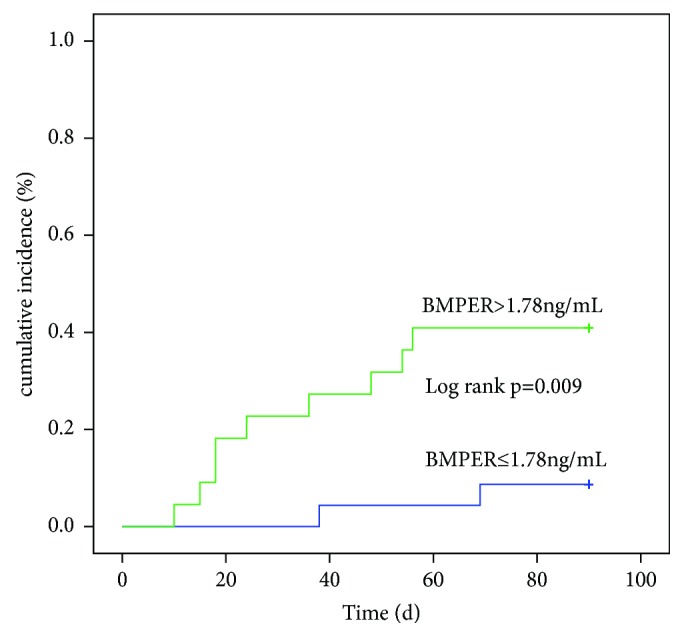
Cumulative incidence of the adverse events for patients with urosepsis that were subdivided into two groups according to the BMPER cut-off value. BMPER: BMP-binding endothelial cell precursor-derived regulator.

All sepsis patients were divided into two groups according to the median BMPER level. After the 90 day follow-up, the cumulative incidence of the adverse events was 8.7% (2/23) for patients below the cut-off value. Two patients were readmitted to hospital, whereas the cumulative incidence of adverse events was 40.9% (9/22) for patients above the cut-off value (Figure 3), including three deaths and six readmissions. The difference in cumulative incidence rates between the groups above and below the cut-off values by log-rank test was significant.

## 4. Discussion

URSL is a widely used method for ureteral stones treatment and poses a risk for postoperative urosepsis [14]. Endothelial inflammation initiating multiorgan failure is critical for patients' outcome in sepsis [10]. BMPER acts as a key regulator in endothelial biology [9]. Our study showed that BMPER is an essential biomarker in urosepsis. This primary conclusion was supported by several key observations. First, compared with control individuals, BMPER level was increased in uroseptic patients and in proportional to the irrigation pressure. Second, the BMPER level was associated with the serum LAC concentration and SOFA score. Third, using SOFA as the gold standard, the diagnostic value of BMPER for urosepsis was acceptable when comparing with other indicators. Fourth, BMPER was a strong predictor of adverse outcome in patients with urosepsis. Hence, these findings provide significant insights into our understanding of the diagnostic and prognostic values of BMPER in urosepsis.

The incidence of lethal post-URSL infection is no longer negligible, but only a limited number of studies have focused on the risk factors associated with post-URSL infection under the Sepsis 3 definition [15]. In the current study, the uroseptic rates were proportional to the irrigation pressure by univariate analysis. In the logistic regression analysis, operation time, DM, and irrigation pressure were the independent risk factors for urosepsis. Patients who underwent ureteroscopy with high irrigation pressure possessed a significantly high risk of urinary sepsis after surgery, as indicated by its high OR (OR = 3.410, 95% CI, and 1.262 to 9.212). Therefore, this result may strengthen the importance of performing URSL under low irrigation pressure in clinical practice. DM and operation time are the possible risk factors for postoperative infections [15, 16]. This result was consistent with our study and showed that operation time and DM were also two independent risk factors for urosepsis. Hence, controlling the operation time for patients with DM is important.

On the 1st postoperative day, laboratory indicators were significantly different between patients with urosepsis and those who did not have for all patients who underwent URSL. For patients with sepsis, BMPER concentrations were increased and associated with CRP, PCT, LAC, and SOFA. By incorporating these four variables into a multiple linear regression model, we found that LAC and SOFA were significantly associated with BMPER. On one hand, multiple factors, such as insufficient tissue oxygen delivery, impaired aerobic respiration, accelerated aerobic glycolysis, and reduced hepatic clearance, lead to increased LAC level. Hyperlactatemia is reflective of cellular dysfunction in sepsis [17]. On the other hand, the Sepsis 3 definitions are based on SOFA and a new scoring system. Fukushima et al. [18] showed that excellent SOFA performance for mortality prediction in patients with acute pyelonephritis is caused by stone obstruction. Therefore, LAC may reflect cellular dysfunction and SOFA can reflect organ dysfunction [17]. BMPER may be associated with dysfunction at cellular and organ levels to a certain extent.

Biomarkers play important roles in the diagnosis and prognosis judgement in sepsis. PCT is a marker of systemic inflammation and aids urosepsis diagnosis [19]. A study revealed that the PCT level can be misleading due to its sensitivity in critically ill patients [20]. CRP was also used to diagnose infection in previous studies [21]. The present study showed that the older biomarkers of WBC and serum CRP and PCT concentrations were good diagnostic indicators of urosepsis according to their AUCs, which agreed with the results of previous reports. The diagnostic value of PCT was still higher than those of the other biomarkers. Given that host genetics is diverse, the processes related to response to infection can be highly different from person to person. Therefore, the combination of different biomarkers facilitates the clinician's diagnostic decisions [22]. PCT, combined with BMPER, has significantly higher diagnostic value than any of the single indicators for urosepsis.

Several clinical studies have demonstrated that different biomarkers are predictors of adverse outcome in patients with sepsis. Previous studies showed that endothelial cell-specific molecule-1/endocan, presepsin, and pentraxin-3 possessed good prognostic value, but these studies were conducted in the intensive care unit [23–25]. A previous study reported the prognostic value of SOFA in patients with urosepsis and in-hospital mortality and intensive care unit (ICU) admission were used as outcomes; this process was different from the procedure used in our study [18]. To our knowledge, a study focusing on the prognostic value of BMPER in patients with urosepsis has not been conducted. Our results suggested that patients with high serum BMPER concentration had high mortality or readmission rate due to febrile urinary tract infection. These results demonstrated that BMPER is a strong predictor of adverse outcome in patients with urosepsis.

This study has several limitations. First, a previous study reported that 7.4% of the patients had an episode of urosepsis after URSL, and the urosepsis rate in our study was higher than that in that study [15]. Patients' selection bias may be the main reason for our result. For some patients with ureteral calculi, nephrostomy or ureteral stent was used for drainage in the first session, and lithotripsy was conducted in the second session. These patients were excluded because they failed to meet the inclusion criteria. Second, this work is a retrospective study; thus, some potential bias and confounding factors are inevitable. Further prospective multicenter study may verify the results of the present study.

## 5. Conclusions

Our study showed that BMPER is related to cellular and organ dysfunction in patients with urosepsis and revealed valuable discriminative capacity for urosepsis. BMPER is also a strong predictor of adverse outcome in patients with urosepsis according to the latest Sepsis-3 definitions.

## Figures and Tables

**Table 1:** Baseline characteristics of the study subjects.

Variable	Control (n=85)	UUO (n=305)			*P* value
		Group I (n=112)	Group II (n=103)	Group III (n=90)	
Age (years)	49(46,57)	48(44,57)	52(47,57)	53(46,58)	0.162
Gender					0.941
Male	32	44	43	37	
Female	53	68	60	53	
DM (n)	8	13	10	9	0.955
BMI	22.0(19.9,24.5)	21.9(20.3,23.4)	21.8(19.9,23.1)	22.1(20.5,23.5)	0.663
Laterality					0.854
Left		60	52	45	
Right		52	51	45	
Stone site					0.895
Proximal		37	34	29	
Middle		28	28	28	
Distal		50	41	33	
Stone size (mm)		7.5(6.4,8.6)	7.7(6.4,8.5)	7.8(6.7,8.5)	0.451
Hydronephrosis (mm)		2.3 ± 0.6	2.1 ± 0.5	2.2 ± 0.4	0.122
Operation time (min)		25(21,27)	25(22,29)	25(22,28)	0.187
WBC (10^9^/L)	7.3 ± 1.6	7.3 ± 1.8	6.9 ± 1.5	7.5 ± 1.8	0.130
T	36.6(36.5,36.7)	36.6(36.5,36.7)	36.6(36.5,36.7)	36.7(36.5,36.8)	0.329
HR	78 ± 15	74 ± 15	74 ± 15	78 ± 16	0.085
MAP	94(86,100)	91(81,101)	96(84,103)	94(82,101)	0.433
PLT	171(152,198)	186(157,212)	190(156,208)	178(156,201)	0.169
CRP	5(5,6)	5(4,6)	5(5,6)	6(5,6)	0.272
PCT	0.21 ± 0.10	0.23 ± 0.13	0.22 ± 0.13	0.25 ± 0.12	0.086
BMPER	0.79 ± 0.17	0.84 ± 0.30	0.78 ± 0.21	0.80 ± 0.17	0.359
Cr	73.2 ± 9.3	73.6 ± 11.4	73.2 ± 11.3	72.9 ± 11.9	0.980
Serum albumin	37.6 ± 1.4	37.6 ± 1.3	37.3 ± 1.4	37.5 ± 1.1	0.187
Bilirubin	10.78(7.38,12.65)	11.49(8.88,14.10)	11.06(8.60,14.29)	10.63(8.57,13.84)	0.322

UUO: unilateral ureteral obstruction; DM: diabetes mellitus; BMI: body mass index; WBC: white blood cell; T: temperature; HR: heart rate; MAP: mean arterial pressure; PLT: platelet; CRP: C-reactive protein; PCT: procalcitonin; BMPER: BMP-binding endothelial cell precursor-derived regulator; Cr: creatinine. Group I: low irrigation group; Group II: medium irrigation group; Group III: high irrigation group.

The normal values are as follows: WBC: 4.0-10.0×10^9^/L; T: 36.0-37.0°C; HR: 60-100/min; MAP: 70~105 mmHg; CRP: 0~10 mg/L; PCT: 0~0.5 ng/mL; Cr: 44-133 *μ*mol/L; serum albumin: 35-40 g/L; Bilirubin: 1.71-21 *μ*mol/L.

**Table 2:** Parameters of patients with urosepsis 1 day after URSL.

Variable	Non urosepsis	Urosepsis	Uroseptic	*P *value
	(n=260)	(n=31)	Shock (n=14)	
Age (years)	51.2 ± 7.5	51.7 ± 7.8	52.9 ± 5.1	0.735*∗*
Gender				0.535
Male	126	13	5	
Female	134	18	9	
DM (n)	17	11	4	<0.001
BMI	22.1 ± 6.4	23.4 ± 4.4	23.3 ± 4.0	0.518*∗*
Laterality				0.861
Left	134	15	8	
Right	126	16	6	
Stone site				0.137
Proximal	83	10	8	
Middle	76	5	2	
Distal	100	16	4	
Stone size (mm)	7.6 ± 2.4	7.9 ± 3.7	7.7 ± 2.1	0.610
Hydronephrosis (cm)	2.1 ± 0.3	1.9 ± 0.4	2.3 ± 0.3	0.071
Irrigation pressure				0.006
Low-grade	104	7	1	
Medium-grade	88	11	4	
High-grade	68	13	10	
Operation time (min)	23.7 ± 5.3	26.3 ± 4.0	29.0 ± 3.9	<0.001*∗*
WBC (10^9^/L)	12.6 ± 3.1	15.4 ± 2.9	17.5 ± 3.9	<0.001
T	36.5 ± 0.08	39.2 ± 0.2	39.2 ± 0.2	<0.001*∗*
HR	77 ± 16	94 ± 8	92 ± 9	<0.001*∗*
MAP	90(80,101)	77(72,86)	64(60,66)	<0.001*∗*
PiO_2_/FiO_2_	402 ± 9	371 ± 56	359 ± 36	<0.001*∗*
PLT	213 ± 32	161 ± 51	145 ± 34	<0.001*∗*
CRP	12 ± 5	14 ± 2	24 ± 4	<0.001*∗*
PCT	1.32 ± 0.61	2.33 ± 1.14	3.60 ± 1.19	<0.001*∗*
BMPER	1.23 ± 0.52	1.62 ± 0.24	3.53 ± 0.77	<0.001*∗*
Cr	76 ± 16	154 ± 35	173 ± 33	<0.001*∗*
Serum albumin	37.3 ± 1.9	31.9 ± 3.2	31.6 ± 3.3	<0.001*∗*
Bilirubin	11.44(9.83,12.92)	22.68(16.04,29.22)	24.91(19.89,31.10)	<0.001*∗*
LAC	1.5(1.3,1.5)	1.6(1.4,1.8)	2.2(2.1,2.3)	<0.001*∗*

*∗*Kruskal-Wallis test. URSL: ureteroscopic lithotripsy; DM: diabetes mellitus; BMI: body mass index; T: temperature; HR: heart rate; MAP: mean arterial pressure; PLT: platelet; CRP: C-reactive protein; PCT: procalcitonin; BMPER: BMP-binding endothelial cell precursor-derived regulator; Cr: creatinine; LAC: lactate.

**Table 3:** Logistic regression analysis of significant factors for urosepsis.

	B	S.E	Wals	df	Sig	Exp(B)	95% CI	
							lower	upper
Operation time	0.121	0.041	8.744	1	0.003	1.129	1.042	1.223
DM	2.139	0.445	23.117	1	0.000	8.487	3.549	20.294
Group I			7.030	2	0.030			
Group II	0.475	0.529	0.808	1	0.369	1.609	0.570	4.537
Group III	1.227	0.507	5.852	1	0.016	3.410	1.262	9.212
constant	-5.837	1.064	30.075	1	0.000	0.003		

DM: diabetes mellitus; Group I: low irrigation group; Group II: medium irrigation group; Group III: high irrigation group.

**Table 4:** Univariate correlations of BMPER with laboratory parameters in patients with urosepsis at 1 day after URSL.

	*r*	*P *value
WBC (10^9^/L)	0.171	0.261
PLT	-0.197	0.195
CRP	0.589	0.000
PCT	0.324	0.030
Cr	0.250	0.097
Serum albumin	-0.033	0.830
Bilirubin	0.148	0.332
LAC	0.689	0.000
SOFA	0.568	0.000

WBC: white blood cell; PLT: platelet; CRP: C-reactive protein; PCT: procalcitonin; BMPER: BMP-binding endothelial cell precursor-derived regulator r; Cr: creatinine; LAC: lactate; SOFA: sequential organ failure assessment.

**Table 5:** Stepwise multiple linear regression analysis of significant factors for BMPER.

	B	S.E	t	sig	95%CI for B	
					lower	upper
constant	-1.228	0.450	-2.727	0.009	-2.137	-0.319
LAC	1.371	0.306	4.479	0.000	0.753	1.988
SOFA	0.202	0.061	3.336	0.002	0.080	0.324

LAC: lactate; SOFA: sequential organ failure assessment.

**Table 6:** The AUC values of WBC, CRP, LAC, PCT, BMPER and PCT combined with BMPER for URSL-induced urosepsis diagnosis.

Variables	Area	SE	P	95%CI	
				lower	upper
WBC	0.762	0.037	0.000	0.690	0.835
CRP	0.784	0.032	0.000	0.721	0.847
PCT	0.843	0.041	0.000	0.762	0.923
BMPER	0.829	0.028	0.000	0.773	0.884
PCT+BMPER	0.901	0.030	0.000	0.843	0.959

WBC: white blood cell; CRP: C-reactive protein; PCT: procalcitonin; BMPER: BMP-binding endothelial cell precursor-derived regulator; URSL: ureteroscopic lithotripsy.

## Data Availability

The datasets used in the current study are available from the corresponding author on reasonable request.
